# A medium-term follow-up of adult lumbar tuberculosis treating with 3 surgical approaches

**DOI:** 10.1097/MD.0000000000008574

**Published:** 2017-11-10

**Authors:** Qi Hong Zhang, Qiang Guo, Chaofeng Guo, Jianhuang Wu, Jinyang Liu, Qile Gao, Yuxiang Wang

**Affiliations:** Department of Spine Surgery, Xiangya Hospital of Central South University, Changsha, Hunan, China.

**Keywords:** anterior approach only, combined posterior and anterior approach, complication, debridement, fusion, instrumentation, lumbar spinal tuberculosis, outcomes, posterior approach only, surgical management

## Abstract

Surgical intervention is an important option for treating lumbar tuberculosis. Previous studies have reported different surgical intervention procedures. To our knowledge, few studies have compared the clinical results of mid-term follow-up of 3 different surgeries in surgical treatment of spinal tuberculosis. This study's purpose is to evaluate the effectiveness of 3 different surgeries for the treatment of lumbar tuberculosis in adult and analyze the mid-term influence of the surgery on quality of life.

Between June 2004 and January 2010, a total of 137 adult patients (54 women and 83 men) with lumbar tuberculosis were recruited for this study. The patients were divided into 3 groups based on administered surgeries: posterior, anterior, and combined posterior-anterior. The trauma index (operation time, blood loss, length of hospital stay, and complications), imaging parameters (segment kyphotic angle, correction rate, loss angle, and bone fusion time), and quality-of-life indicators, including Oswestry Disability Index (ODI), the Frankel grade, visual analog scale (VAS), and Macnab score, were collected.

The posterior group experienced the lowest trauma index, whereas the combined group faced the highest trauma index. The anterior group's kyphosis correction rate of (52% ± 5.45%) was significantly inferior to the posterior group (74% ± 5.04%) and the combined group (69% ± 7.95%), whereas the loss of correction in the anterior group (2.5°) was higher than the losses of correction in the posterior group (0.8°) and combined group (1.1°). The mean bone fusion times of the 3 groups were similar. Postsurgery quality of life was markedly improved in all patients. The improvement rates of the ODI, VAS, and the excellent and good rate per the Macnab score were similar among the 3 groups at the final follow-up.

Based on a retrospective study, for patients with lumbar tuberculosis, use of the anterior approach should be limited. Although the combined approach produced satisfactory outcomes, it remains more traumatic. Compared with the anterior surgery and the combined surgery, the posterior-only approach is safer and less invasive.

## 1 Introduction

Spinal tuberculosis (TB), caused by *Mycobacterium tuberculosis*, is still a challenging hazard in the world.^[1]^ Spinal TB, accounting for a large part of osteoarticular TB, most commonly causes lumbar destruction with severe complications in adults.^[2]^ Even though lumbar TB is generally treated with drug therapy, the drug cannot easily cure “closed lesion.” Worse, 8% to 12% of the patients have resistance to first-line antitubercular drugs.^[3]^ Surgical intervention is needed in patients who have existing or residual deformity, severe neurologic dysfunction, or extensive abscess to help cure the infection, prevent the spinal deformity progression, and prevent the worsening of neurologic deficits. At the same time, surgical intervention can remove the sclerotic wall, opening penetration of the anti-TB drugs into the foci.

Three main surgical strategies treating lumbar TB exist: the posterior-only approach, the anterior-only approach, and the combined approach (combined anterior and posterior approach).^[4]^ The anterior and the combined approaches have been widely used by researchers to effectively treat lumbar TB. However, the anterior approach generally creates more complications, and the combined approach leads to longer recovery times as a result of 2 incisions.^[5]^ In recent years, surgeons have become increasingly accepting of the posterior-only approach. Zhang et al reported that the posterior-only surgery involving focus debridement, interbody graft, posterior instrumentation, and fusion is an effective treatment for lumbar TB that leads to satisfactory short-term outcomes with a minimum amount of trauma.^[6–8]^

Surgical choice is still controversial. More research is required to determine the optimal surgical approach to treat lumbar TB individually and effectively. Mid-term outcomes of different surgeries for the treatment of lumbar TB in adults has scarcely been reported. Therefore, we conducted a retrospective evaluation of 137 cases of lumbar TB with different surgeries to provide further clinical guidance on selecting surgical treatments.

## 2 Materials and methods

### 2.1 Inclusion and exclusion criteria

#### 2.1.1 Inclusion criteria

The lesions were confined to 1 segment or 2 adjacent segments without extensive abscess (or if multiple segments were involved, only 1 or 2 vertebral bodies needed to be addressed surgically). Additionally, 1 or more of the following situations were present in our patients: spinal instability or vertebral collapse resulted from vertebral damage; obvious or ongoing kyphosis; severe or progressive spinal cord functional damage by the compression of abscess or necrosis existed; or significant bone necrosis or sequestrum. All studied patients had at least 5 years of complete follow-up data.

#### 2.1.2 Exclusion criteria

Patients presenting the following conditions were excluded from surgery: multilevel lumbar spinal TB with a large paravertebral stream-like abscess; severe osteoporosis or multiple organ dysfunctions or active pulmonary TB, which made the patient unable to tolerate the surgery; a history of lumbar surgery, congenital spinal deformity, or other medical history that would influence postoperative evaluation.

### 2.2 Patients

This study was approved by the Ethics Board Committee of our hospital. From June 2004 to January 2010, 137 lumbar TB cases in our hospital were enrolled in the study, including 83 males and 54 females. Among these patients, 63 cases were treated with debridement, interbody fusion, and internal fixation via posterior approach only; 32 cases were treated with anterior debridement and strut grafting with instrumentation; and 42 cases were treated with posterior fixation, anterior debridement, and bone graft in single or 2-stage procedures. In clinical practice, it is not possible to randomly select a surgical treatment method. Therefore, we did the anterior-only approach in an earlier period compared with the other 2 approaches in our study. Written informed consent was acquired from each of the patients to authorize treatment, image findings, and photographic documentation. All surgical procedures were performed by the same surgeons at the same institution.

Patients were aged from 20 to 75 years, with a mean of (65.6 ± 15.5) years. The duration of symptoms was from 2 to 15 months, with an average of 8.3 ± 4.2 months. All patients suffered from varying degrees of back pain and fatigue. Forty-eight patients (35.0%) experienced fever and night sweats; 87 patients (63.5%) had varying degrees of leg pain. The symptom of spinal nerve damage was found in 121 (88%) patients. According to Frankel grade, patients were rated as grade B, 23 patients; grade C, 30 patients; and grade D, 68 patients. All patients received preoperative x-ray, computed tomography (CT) scan, and magnetic resonance examinations. Imaging showed vertebral destruction in 106 patients (77.4%), bone necrosis in 121 cases (88.3%), paravertebral abscess in 62 cases (45.3%), intervertebral space narrowing in 106 cases (77.4%), and kyphosis in 95 cases (69.3%). Clinical details of the patients and segment involved distribution are presented in Table 1.

**Table 1:**
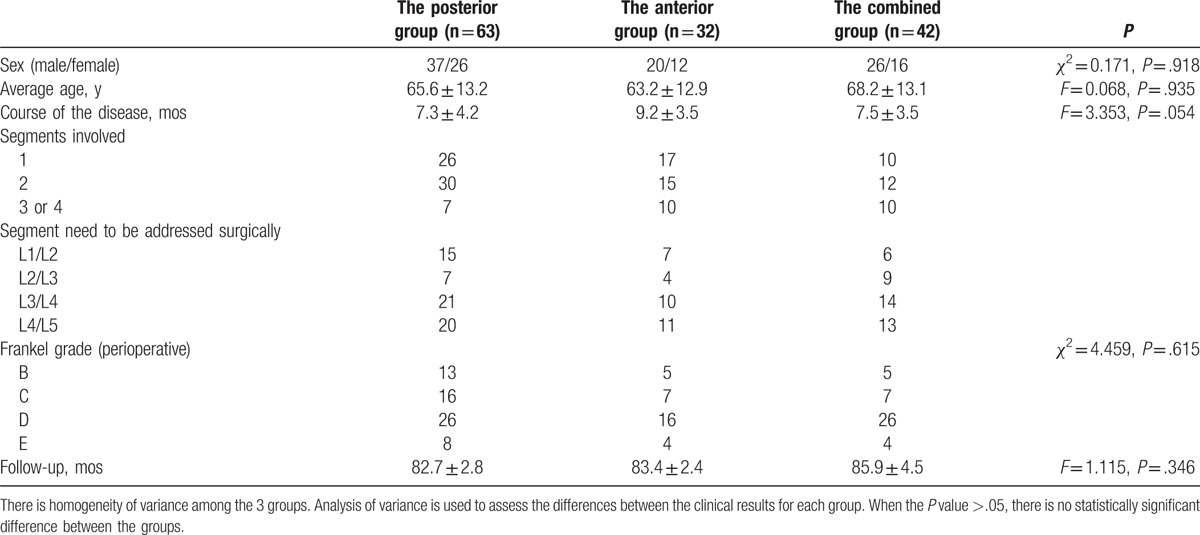
Clinical data of patients.

### 2.3 Methods

#### 2.3.1 Preoperative procedure

Patients’ knowledge regarding spinal TB recovery was reinforced after the patients were hospitalized. They were asked to take strict bed rest and given nutritional advice. The initial anti-TB treatment consisted of isoniazid (5 mg/kg; maximum 300 mg/d), rifampicin (15 mg/kg; maximum 600 mg/d), ethambutol (15–25 mg/kg; maximum 2 g/d), and pyrazinamide (15–30 mg/kg; maximum 2 g/d). This medication was used for at least 4 to 6 weeks before operation. When the related indicator of inflammation, such as erythrocyte sedimentation rate and C-reactive protein, had significantly decreased and TB clinical symptoms were relieved, the surgery was performed. If a patient experienced paralysis during presurgical chemotherapy, the surgery was performed even if the indicator did not decrease. The same surgeon reviewed all surgical indications and performed the procedures.

#### 2.3.2 Surgical procedure

**Figure 1:**
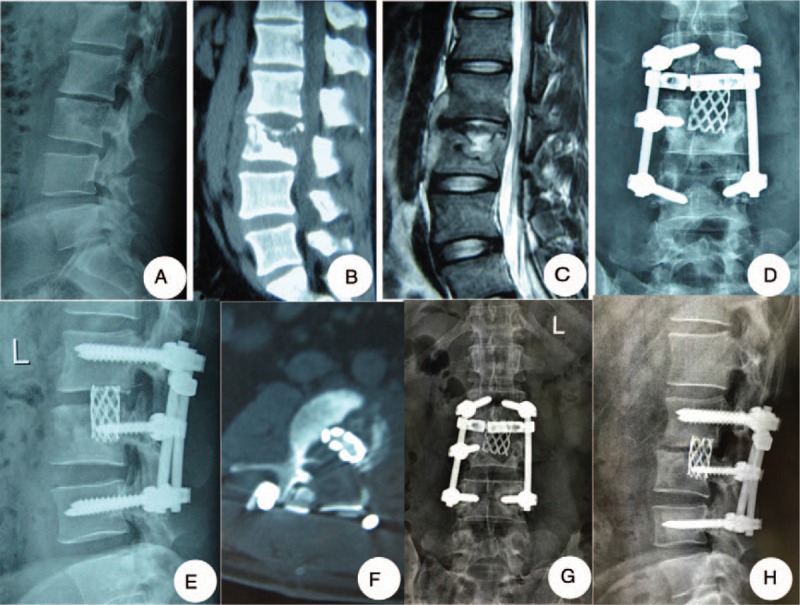
One patient in the posterior group: a 24-year-old man of whom lumbar tuberculosis at L2-3 was diagnosed underwent debridement, interbody fusion and internal fixation via posterior approach only. (A–C) Preoperative images showed that the lesion around the vertebra body of L2/3 developed an abscess with marked bony destruction. (D, E) Postoperative x-ray showed good internal fixation. (F) Postoperative CT showed interbody graft using titanium mesh cages were placed satisfactorily. (G, H) Six-year final follow-up x-ray showed good bone fusion, and no evidence of instrumentation failure was found.

In the posterior group, under general anesthesia, with somatosensory-evoked potential monitoring, and in the prone position, surgery was performed. The lamina, facet joints, and transverse processes were exposed with a posterior median incision. It is necessary to dissect the above extraperiosteal to reduce blood loss. Screws were planted through the vertebral arch. Shorter segmental fixation, at least 1 above and 1 below the lesion, was preferred. Screws were placed in the destroyed vertebrae if possible and the upper part of the vertebrae had not eroded. One rod was temporarily fixed to the mildly affected side of the lesion before the decompression and focal debridement to avoid spinal cord injury. The prevertebral abscess was drained and the focus exposed from the more severely affected side of the lesion segment. Different sizes of spatulas and angles were used to remove the lesions, including sequestra, abscess, and granulation tissue, as completely as possible. The abscess was drained by suction and curettage as thoroughly as possible. The grafts consisted of trimmed allograft bone or titanium mesh cages as appropriate. One or 2 titanium mesh cages which were filled with autogenous bone from the healthy lamina, spinous process, and allograft bone were fashioned according to the shape and length of bone graft bed. After the deformity was corrected, both side rods were fixed and both sides compressed to tighten the mesh cages and bone blocks. Then, for all patients, the strips of autogenous bone or allograft were implanted for posterior reconstruction on the segments that had undergone decompression. Local administration of 1.0 g streptomycin and 0.3 g isoniazid was given. The drainage and incision sutures were carefully performed (Fig. 1).

**Figure 2:**
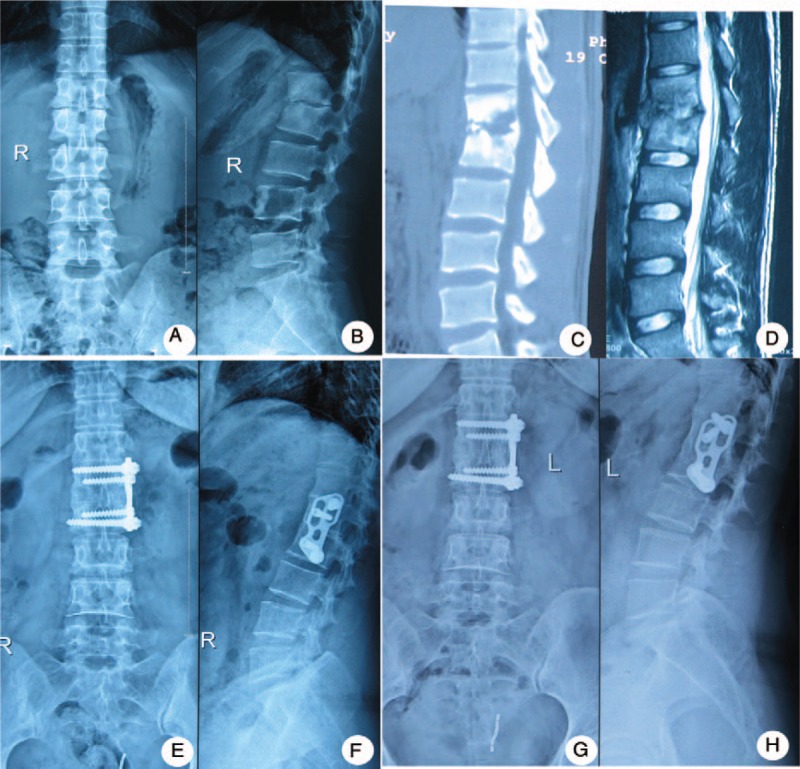
One patient in the anterior group: a 40-year-old woman was diagnosed as having lumbar tuberculosis (L1-2) who was treated with the anterior debridement and strut grafting with instrumentation. (A–D) The destructive changes, including narrowing of intervertebral space, sequestrum, seen at L1/2 on plain radiographs and CT, were progressive and paravertebral abscess was shown on MRI. (E, F) Postoperative x-ray showed good internal fixation and disappearance of the segmental instability. (G, H) 11-year-follow-up x-ray showed formation of bridging osteophytes and no flexible internal fixation or ruptures. MRI = magnetic resonance imaging.

In the anterior group, oblique hypogastric incisions were made into patients’ TB foci. After retroperitoneal exposure, the foci were removed completely from the anterior. We used appropriate trimmed allograft bone block for the bone graft. The pedicle screws and rod system were placed in adjacent vertebrae to fix the bone and correct the kyphosis (Fig. 2).

**Figure 3:**
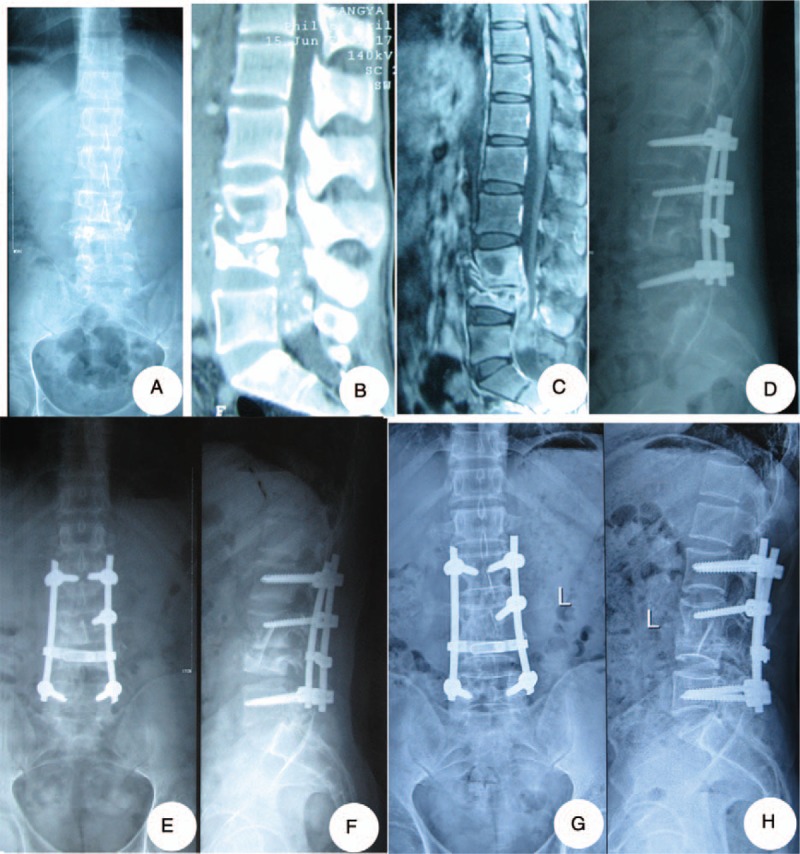
One patient in the combined group: a-26-year-old man who suffered from L3-4 spinal tuberculosis received posterior fixation, anterior debridement, and bone graft in single or 2-stage procedures. (A–C) The case had collapse of vertebral body, the segmental kyphosis of lumbar, compression of spinal cord due to the psoas abscess. (D) Postoperative x-ray showed that the lumbar lordosis was restored with good location of both bone grafting and internal fixation device. (E, F) One-year final follow-up x-ray showed good bone fusion. (G, H) The x-ray films showed that there was no instability or looseness of the internal fixation at 6 years.

In the combined group, first, via the posterior approach, the pedicle screw-rod internal fixation was applied. Then, anterior debridement and interbody fusion were applied in the single or 2-stage operation. Autologous bones or allograft bones were implanted in the defects (Fig. 3).

### 2.4 Postoperative treatment

Postoperative treatments were applied according to the patients’ conditions. With external braces, patients were encouraged to take functional ground exercise as early as possible. Anti-TB treatments were continued for 12 to 18 months. Drug side effects were closely monitored, and medications were adjusted based on the follow-up data until patients completed their medication regimens.

### 2.5 Outcomes

All patients were examined clinically and radiologically at 3, 6, and 12 months after surgery, and once a year thereafter. Patients’ data consisted of the trauma index (operation time, blood loss, length of hospital stay, complications), imaging parameters (segment kyphotic angle, corrective rate, loss angle, bone fusion time), and quality-of-life indicators (Oswestry Disability Index [ODI], the Frankel grade, visual analogue scale [VAS], Macnab score). An independent, experienced spine surgery specialist performed the clinical evaluations. The follow-up was done through the postal questionnaire, telephone consultation, or outpatient visits.

### 2.6 Statistical analysis

Results were recorded and analyzed using SPSS software version 22.0 (SPSS Inc., Chicago, IL). Operative times, blood loss, mean fusion times, and kyphosis angles were statistically analyzed using analysis of variance first and then by using the Student-Newman-Keuls test to compare each group. The measurement data in the 3 groups was statistically analyzed using the chi-square test first and then by partitioning the chi-square test to compare each group. Discrepancies in the normal distribution were analyzed by a rank-sum test with a significance level of 0.05.

### 2.7 Ethics approval

The Ethics Committee in Xiangya Hospital of Central South University provided the ethics approval.

## 3 Results

### 3.1 Clinical data

There were no significant differences between the three groups in terms of the patients’ sex, age, course of the disease, follow-up time (*P* > .05, Table 1,); 121 patients had neurological impairment, including 55 cases in the posterior group, 38 in the combined anterior-posterior group, and 28 in the anterior group. The differences among the 3 groups were not statistically significant (*P* > .05, Table 1), indicating that the data of the 3 groups of patients are comparable.

### 3.2 Injury indicators

For the posterior group, the mean operation time and average blood loss were 207.9 ± 30.9 minutes and 409.5 ± 107.9 mL, respectively; for the anterior group, they were 270.7 ± 32.0 minutes and 649.0 ± 120.0 mL; for the combined anterior-posterior group, they were 349.7 ± 38.9 minutes and 840.0 ± 168.7 mL. Both the mean operation time and the average blood loss of the posterior group were less than those of the anterior group and those of the combined anterior-posterior group. Both the mean operation time and the average blood loss of the combined group were more than those of the anterior group (*P* < .05, Table 2). The average lengths of hospital stay for the 3 groups of patients were 13 ± 3.2, 14 ± 2.4, and 19 ± 2.1 days, respectively. The differences between the 3 groups were statistically significant. The stay lengths of the anterior and the posterior groups were shorter than the combined group (*P* < .05), and there was no statistically significant difference between the anterior group and the posterior group (*P* > .05, Table 2).

**Table 2:**
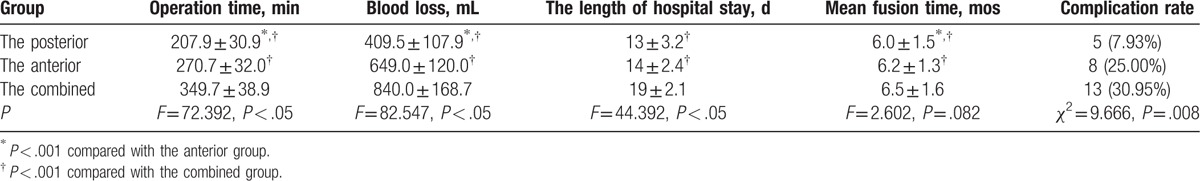
Injury indicators of the 3 surgical groups for lumbar spinal tuberculosis.

### 3.3 Radiological indicators

The fusion times for the posterior group, anterior group, and combined anterior-posterior groups were 6.0 ± 1.5, 6.2 ± 1.3, and 6.5 ± 1.6 months, respectively, and there was no statistically significant difference (*P* > .05, Table 2). The final follow-up imaging examination showed complete bone fusion (15), no bone nonunion, no pseudarthrosis or loosening, and no fracturing (Figs. 1–3).

In the posterior group, the postoperative kyphosis angle correction rate was 74% ± 5.04%, and the correction loss at the final follow-up was 0.8°; in the anterior group, the postoperative kyphosis angle correction rate was 52% ± 5.45% and the correction loss at the final follow-up was 2.5°; in the combined anterior-posterior group, the postoperative kyphosis angle correction rate was 69% ± 7.95% and the corrective angle at the final follow-up was 1.1°. The postoperative kyphosis correction rate and the correction loss at the final follow-up between the posterior group and the combined group showed no statistically significant difference (*P* > .05); the correction rate of the anterior group was lower than that of the other 2 groups (*P* < .05), the correction loss was higher than that of the other 2 groups (*P* < .05, Table 3).

**Table 3:**
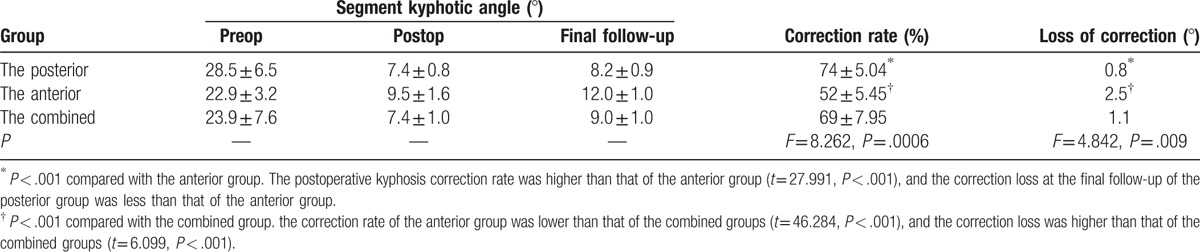
Radiological data of the 3 surgical groups for lumbar spinal tuberculosis.

### 3.4 The quality-of-life index

For patients with preoperative spinal nerve dysfunction symptoms, at the final follow-up, 5 cases in the posterior group were improved from a Frankel grade B to D, and the rest were improved to grade E; 2 cases in the anterior group were improved from a Frankel grade B preoperatively to D, and the rest were improved to grade E; 2 cases in the combined anterior-posterior group were improved from grade C preoperatively to D, and the rest were improved to grade E. There was no statistically significant difference in spinal cord function improvement among the 3 groups (*P* > .05, Table 4).

**Table 4:**
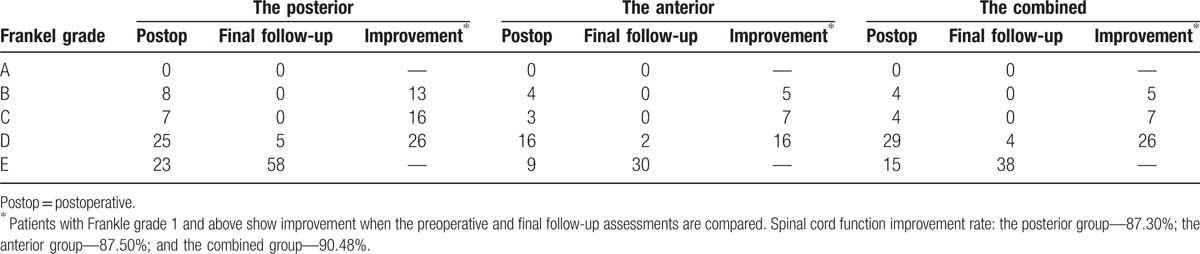
The improvement of the Frankle grade of the 3 surgical groups for lumbar spinal tuberculosis.

At the final follow-up, the ODI and VAS in the posterior group were improved from 36.32 ± 6.76 preoperatively, 8.47 ± 0.72 to 7.05 ± 1.08 points, and 1.55 ± 0.83 points. The ODI and VAS in the anterior group were improved from 36.30 ± 7.41 points, 8.35 ± 0.85 to 8.85 ± 1.30 points, and 1.95 ± 0.82 points. The ODI and VAS in the combined group were improved from 35.94 ± 6.91 points, 8.38 ± 0.54 to 7.54 ± 1.24 points, and 1.64 ± 0.42 points. The differences were not statistically significant (Tables 5 and 6) between the 3 groups. The differences in the ODI and VAS improvement among the 3 groups were not statistically significant (*P* > .05, Table 7). No statistically significant difference was found in the Macnab excellent/good rate among the 3 groups (*P* > .05, Table 7).

**Table 5:**
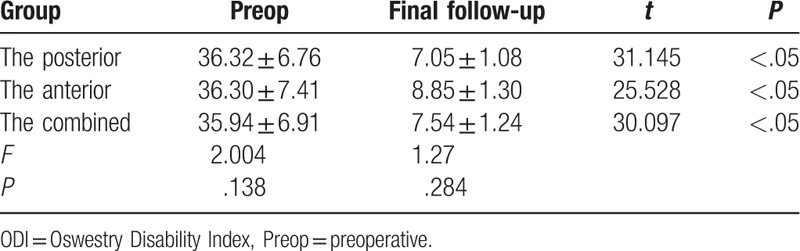
The ODI index of the quality of life (%)of the 3 surgical groups for lumbar spinal tuberculosis.

**Table 6:**
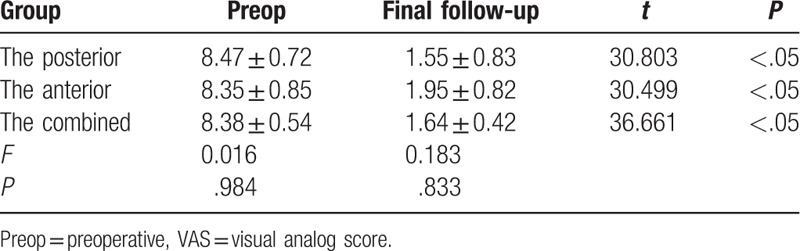
The VAS of the quality of life of the 3 surgical groups for lumbar spinal tuberculosis.

**Table 7:**
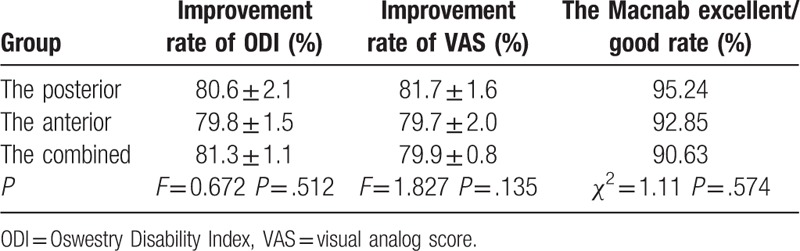
Comparsion of the quality-of-life indicators of the 3 groups.

### 3.5 Complications

In our study, a total of 26 cases of complications were noted in 137 patients. Five cases involved dural tear and intraoperative cerebrospinal fluid leak caused by intraoperative separation of spinal dura mater. Those patients were asked to lie supine, and drainage tubes were attached for an average of 3 days and then removed, with no occurrences of infectious meningitis. One case presented progressive aggravated neurological dysfunction after surgery, classified as epidural hematoma according to the radiography, and after 1 more surgical removal, the patient's nerve function recovered to the preoperative level. One case had abnormal liver functions after 3 months of anti-TB therapy. For this patient, after the treatment was altered from rifampin to rifapentine and the liver-protecting therapy was strengthened, no severe liver malfunction occurred. Six cases involved postoperative ileus, and after routine nasogastric decompression treatments, the patients were relieved. Four cases involved postoperative infection, and after antibiotic treatment, the patients recovered. Three cases involved a fistula within 2 months after surgery, and the patients recovered after CT-guided percutaneous drainage with local injection of anti-TB drugs. Four cases had recurrent abscesses, and after treating with debridement, their mycobacterial TB culture results suggested multidrug-resistant TB, after treating with the recommended medication which was based on the drug susceptibility test results. No recurrence was found during the period between drug withdrawal and the final follow-up. Two patients returned to weight-bearing work after their discharge from the hospital, and at the follow-up, pseudarthrosis and internal fixation loosening were found. They were cured after renovations.

The posterior group had complications in 5 cases, with an incidence rate of 12.95%; the anterior group had complications in 8 cases, with an incidence rate of 25%; the combined group had complications in 13 cases, with an incidence rate of 30.95%. The differences in the incidence rates among the 3 groups were statistically significant (*P* < .05). The posterior group had a lower complication rate than that of the anterior group, and the anterior group had a lower complication rate than that of the combined group (Table 2).

## 4 Discussion

In anti-TB treatment, surgical intervention is a powerful tool, along with medication, for improving patient recover. Surgical intervention can be used to clear the focus, decompress the spinal canal, and reconstruct the spine. Yet, the optimal surgical management method remains under debate. We believe the results of this study should help build a greater consensus around the superiority of the posterior method.

The trauma indicators suggest that the posterior debridement and interbody fusion with instrumentation surgery was less invasive and more efficient compared with traditional surgical treatments (anterior and combined). Yang et al^[5]^ have used the meta-analysis to compare clinical efficacy and safety among 3 surgical approaches for the treatment of spinal TB, and concluded that the posterior approach has better clinical outcomes than anterior or combined approach for spinal TB. The anterior surgical approach enables direct removal of the TB abscess and nerve decompression; however, this approach can cause greater damage to the anterior and middle of the lumbar column and disturb organ function in front of the lumbar, which can complicate postoperative recovery.^[9,10]^ Therefore, anterior surgery is a suboptimal approach for lumbar TB with unserious vertebral damage, such as the mono-segment involved and limited abscess cases. The combined approach requires 2 surgical incisions and sometimes 2 surgeries, creating greater patient trauma. The posterior-only approach gives the surgeon a 270° viewing range of the lumbar canal outside the epidural and an approach to the target area to get complete decompression. It required a relatively simple operation with only 1 incision, In this way, the posterior approach reduced the surgical trauma and produced satisfactory results.

Complications can significantly increase patients’ suffering after surgery,^[11]^ and the incidence rate is considered an important indicator of surgical safety. Some scholars have reported that the anterior approach can significantly increase the risk of complications and even cause serious consequences because of the narrow operation space, extensive blood vessels and nerves, and complex anatomical layers.^[12,13]^ In this study, the incidence of complications in the posterior group was lowest. The posterior approach does not require transection of the anterior muscle tissue or interfere with the anterior kidney, intestine, or large blood vessels, which significantly reduces the incidence of complications, especially serious ones. The posterior approach can clear the focus of infection. In the posterior approach, after focus removal, the drainage tube was directly positioned in the residual sequestrum, allowing the smooth postoperative drainage and significantly reducing complication incidents. When the patient lies supine as in the posterior approach, a good postural drainage forms, benefitting the healing of the nidus. Some surgeons voice concern over the tuberculous meningitis and extension of TB from the anterior lesion to the posterior healthy bone. In our study, TB metastasis did not occur, further demonstrating the relative safety of the posterior-only approach. Most lumbar TB patients suffered significant lower back pain and even neurological damage, greatly impairing their quality of life. The main purpose of surgical treatment is to improve patients’ quality of life utmost. In this study, after surgical treatment, patients with preoperative neurological dysfunction recovered with varying degrees, and there was no significant difference in neurological improvement among the 3 groups of patients.

Spinal TB always occurs in the vertebrae or intervertebral space. Although applying anterior surgery allows the surgeon to directly reach foci and implant bone graft between vertebrae, it cannot prevent kyphosis progress.^[14,15]^ Osteoporosis or osteopenia always affect the involved bone, so firmly fixing the screw and correcting the spinal deformity is challenging. In our study, anterior surgery resulted in the worst correction effects and the most easily lost correction angle. Biomechanical principles allow combining anterior debridement and intervertebral body fusion, and posterior pedicle screw fixation to strengthen the posterior column's stability. The combined approach better corrects kyphosis and can effectively restore the physiological curvature of the spine. In addition, the internal fixation is placed distant from the foci, reducing the possibility of infection and bone nonunion. The posterior group had a higher correction rate and lower loss angle at the mid-term follow-up compared with those of the anterior group, which demonstrates posterior internal fixation's greater efficacy in correcting kyphosis compared with that of the anterior approach. All 3 groups reached complete bone fusion, and there was no statistically significant difference in fusion time. The evidence demonstrates that the posterior approach can realize satisfactory orthopedic effects and not cause spinal instability. We believe that the posterior-only surgery obtained satisfactory orthopedic result for the following reasons: only 1 side of the vertebral plates and intervertebral joints were damaged, limiting the impact on spinal stability; the fixation range was appropriately selected: generally a pair of vertebral pedicle screws was implanted to the first or second healthy upper and lower vertebra above and below the unhealthy vertebra, and transpedicular screws were also placed in the affected vertebrae if the upper part of the vertebrae was not destroyed by infection; intervertebral bone graft and posterior lamina reconstruction was adequate; the appropriate graft was selected to match the shapes of the foci to better fit the bone bed.

Every approach has specific advantages. In practice, we should select the approach that meets the individual needs of treatment. Indications and contraindications must be attended to in clinical application. The anterior-only approach should be avoided in patients with severe kyphosis more than 60°, posterior column damage, and in patients with poor abdomen health.^[16,17]^ For cases involving severe vertebral body destruction (the involved unit is more than 3), challenging anterior fixation, or severe kyphosis, the combined surgery should be suggested. However, we should be cautious using the combined approach for the patients in poor health.^[18,19]^ Based on reviewing the follow-up results, it was found that TB involving 1 spinal functional unit (foci mainly in the intervertebral space; the number of unhealthy intervertebral spaces is fewer than 2) with a not excessively large paraspinal abscess is the most suitable type for applying the posterior-only approach. In addition, the posterior-only approach may not be suitable for patients who have more than 3 damaged vertebrae and with an extensive abscess.^[6,20,21]^

We acknowledge some limitations in this study. A multicenter study with a larger sample size is desirable to further confirm the indication, feasibility, reliability, and complications of the posterior-only approach. In addition, more longitudinal follow-up is required to evaluate whether the instrumentation has a long-term effect on the sagittal imbalance and living quality of patients.

## 5 Conclusions

In conclusion, for patients with lumbar TB, use of the anterior approach should be limited. Although the combined approach produced satisfactory outcomes, it remains more traumatic. Compared with the anterior surgery and the combined surgery, the posterior-only approach is safer and less invasive.
