# A medium-term follow-up of adult lumbar tuberculosis treating with 3 surgical approaches: Erratum

**DOI:** 10.1097/MD.0000000000010039

**Published:** 2018-02-23

In the article, “A medium-term follow-up of adult lumbar tuberculosis treating with 3 surgical approaches”,^[1]^ which appeared in Volume 96, Issue 45 of *Medicine*, Dr. Hongqi Zhang appeared incorrectly as Qi Hong Zhang.
